# Psychometric Properties of the Chinese Version of the Mindfulness in Teaching Scale

**DOI:** 10.3390/ijerph16132405

**Published:** 2019-07-06

**Authors:** Chunxiao Li, Ying Hwa Kee, Yandan Wu

**Affiliations:** 1 Physical Education and Sports Science Academic Group, National Institute of Education, Nanyang Technological University, Singapore 637616, Singapore; 2 School of Physical Education and Sports Science, Fujian Normal University, Fuzhou 350117, China; 3 Department of Health and Physical Education, The Education University of Hong Kong, Hong Kong, China

**Keywords:** validation, reliability, teacher, education, China

## Abstract

Measuring teacher mindfulness has implications for understanding and enhancing teachers’ well-being. This study therefore aimed to examine the psychometric properties of the Chinese version of the Mindfulness in Teaching Scale (MTS-C). Two independent samples (Sample 1 includes 151 in-service teachers, Sample 2 includes 229 pre-service teachers) completed the MTS-C and theoretically relevant measures (i.e., attitudes, self-esteem, self-efficacy, and life satisfaction). In addition, a subsample of Sample 2 completed the MTS-C again one month later. Results of exploratory factor analysis and confirmatory factor analysis supported the two-factor model of the MTS-C. The MTS-C was generally associated with the concurrent measures. Furthermore, the scale also demonstrated good internal consistency and test–retest reliability. These findings suggest that the MTS-C is a reliable and valid tool for research and practical applications among Chinese teachers.

## 1. Introduction

Teachers are vulnerable to experiencing ill-being because of risk factors such as excessive workload, demands from parents and the educational system, and negative interactions with students and colleagues [1,2]. The compromised well-being will affect not only teachers’ mental health, but also their teaching performance [3]. For example, teachers’ emotional problems tend to adversely affect the classroom environment (e.g., creating negative teaching climates) [4]. In addition, highly stressed teachers were found to contribute to impaired executive function and adjustment problems among students [5]. These negative impacts have stimulated the interest in studying teacher well-being [6].

One line of research is to use mindfulness-based approaches to understand and enhance teacher well-being [4,7]. Kabat-Zinn [8] defined mindfulness as “paying attention in a particular way: on purpose, in the present moment and nonjudgmentally” (p. 4). Recent studies have shown that mindfulness is related to teacher well-being and teaching performance. For example, there was a positive relationship between teacher mindfulness and favorable outcomes such as teacher efficacy, job satisfaction, and teaching attitude [6,9]. Emerging evidence is also available regarding the efficacy of mindfulness-based interventions on teacher well-being. For example, mindfulness-based intervention programs were found to decrease anxiety, burnout, sleep problems, and stress among teachers [4,10,11].

Typically, general mindfulness scales such as the Five Facet Mindfulness Questionnaire (FFMQ) [12] and the Mindfulness Attention Awareness Scale (MAAS) [13] have been used in assessing the mindfulness of teachers in the teaching context (for a review, see [4]). However, the lack of mindfulness measures that are designed for use in a specific context (i.e., context specificity) such as parenting and teaching limits advancement in this line of research. Research suggests that context-specific mindfulness measures are more sensitive and useful for discovering domain-specific findings than general mindfulness scales. For example, scores from the Interpersonal Mindfulness in Parenting Scale [14] better predicted infants’ stress levels than that of the FFMQ [15]. In the case of mindfulness measures in sport, Thienot et al. [16] also demonstrated the utility in their Mindfulness Inventory for Sport in terms of its association with conceptually related variables such as flow, worry, concentration disruption, and perfectionism. Works related to the development of context-specific mindfulness measures continue to be undertaken.

As an effort to measure domain-specific mindfulness among teachers, Frank et al. [17] recently developed the Mindfulness in Teaching Scale (MTS), particularly emphasizing the dual notion of a teacher’s intrapersonal and interpersonal mindfulness. In the multi-study validation research of the MTS [17], a pool of 20 items was first developed by experts in mindfulness and educational research in Study 1. The item pool was then subject to an exploratory factor analysis (EFA) with 263 elementary school teachers. In Study 2, the derived two-factor structure from EFA was confirmed using confirmatory factor analysis (CFA) using another sample of 263 elementary school teachers. The finalized MTS consists of 15 items measuring two positively correlated factors, namely, teacher intrapersonal mindfulness (9 items) and teacher interpersonal mindfulness (5 items). Items in the teacher intrapersonal mindfulness subscale measure awareness, attentiveness, and being in the present moment that characterize mindfulness [8]. Items in the teacher interpersonal mindfulness subscale concern an open and receptive awareness during teacher–student interactions. Both subscales demonstrated adequate internal reliability (α ≥ 0.70) in Study 1, and in Study 3 good test–retest reliability across a six-month period (*r* = 0.42 for teacher intrapersonal mindfulness; *r* = 0.49 for teacher interpersonal mindfulness) among 392 kindergarten and elementary school teachers was demonstrated. Study 3 also found that the teacher interpersonal mindfulness subscale positively relates to teacher efficacy and negatively associates with teacher burnout. Overall, these findings attest to the reliability and validity of the MTS.

To extend the utility of the MTS in other contexts, Kim and Singh [6] examined the psychometric properties of the Korean version of the MTS (MTS-K). Two samples comprising kindergarten to high school teachers were subjected to EFA (*n* = 161) and CFA (*n* = 243), respectively. The results of EFA and CFA supported the two-factor model of the MTS. The two subscales had acceptable to good internal reliability (α ≥ 0.61). Moreover, the scores of these two subscales were correlated to theoretically relevant constructs, including dispositional mindfulness, teacher efficacy, teacher burnout, job satisfaction, and job stress. More recently, the psychometric properties of the Turkish version of the MTS (MTS-T) were examined among 409 teachers from kindergarten to high school levels [18]. Their CFA result supported the two-factor model of the MTS-K after removing one item with a low factor loading (< 0.30). The two subscales had acceptable internal reliability (α ≥ 0.64) and test–retest reliability across a period of three weeks (*r* = 0.80 for teacher intrapersonal mindfulness; *r* = 0.64 for teacher interpersonal mindfulness). Moreover, subscale scores had a positive association with the MAAS, supporting the concurrent validity of the MTS-T. These two validation studies generally supported the psychometric properties of the MTS in other contexts.

Although the MTS is a promising scale for teacher mindfulness research and practice, its reliability and validity have not been examined among teachers in China. Given that China has a rich repertoire of mindfulness-related practices [19], the likelihood of mindfulness-related skills being more widely practiced organically may be stronger than in other localities. Such heightened exposure to mindfulness practices, situated within a collectivistic culture, could potentially shape Chinese teacher’s interpretation of mindfulness, both at the intra- and interpersonal levels, in a way that is different from the scale’s original site of development. In support of the continual development of the MTS, the present research was undertaken using two independent samples to examine the psychometric properties of the Chinese translated MTS (MTS-C), including its factorial validity, concurrent validity, internal reliability, and test–retest reliability. Specifically, we (1) explored and confirmed the factor structure (factorial validity) of the MTS-C through factor analyses, (2) examined the concurrent validity of the MTS-C by selecting several theoretically relevant constructs, and (3) determined internal reliability and one-month test–retest reliability of the MTS-C. 

## 2. Materials and Methods 

### 2.1. Participants

Two Chinese samples were recruited from China in this study. Sample 1 consisted of 151 in-service school physical education teachers (male = 84, 55.6%). Half of them were recruited from elementary schools (*n* = 76, 50.3%) and the rest were from secondary schools (*n* = 75, 49.7%). They had a mean age of 34.40 (SD = 8.36, range = 23 to 58) years and taught for 11.21 (SD = 9.51) years. Sample 2 comprised 229 pre-service physical education teachers from five physical education teacher training programs in China. They were required to have part-time teaching experience or have completed their teaching practice to be eligible for this research. Their age ranged from 19 to 27 years (M = 21.42, SD = 1.30) and the majority of them were male (*n* = 171, 74.7%).

### 2.2. Measures

Both samples completed the MTS-C. To examine the concurrent validity of the MTS-C, Sample 1 also completed two standardized scales to measure their attitudes and self-esteem, whereas Sample 2 also reported their teaching self-efficacy and life satisfaction through another two standardized scales. These criterion variables (e.g., attitudes and life satisfaction) were selected as they have been found to relate to mindfulness in previous studies (e.g., [6,18,20]). 

#### 2.2.1. Mindfulness

We used the MTS-C to measure participants’ intrapersonal and interpersonal mindfulness [17]. Two bilingual researchers translated the English version of the MTS into Chinese followed by back translation by another two bilingual researchers [21]. The MTS-C was administered to 10 in-service physical education teachers. Changes to wording were not required by them. The MTS-C consists of 14 items identical to its original version in English (e.g., “When I am in the classroom I have difficulty staying focused on what is happening in the present”). Participants were asked to rate the items on a 5-point Liker scale, ranging from “never true” (1) to “always true” (5). 

#### 2.2.2. Attitudes

We adapted the scale that has been used to measure Chinese teachers’ attitudes toward teaching children with autism spectrum disorder to measure participants’ attitudes towards teaching students with attention deficit and hyperactivity disorder (ADHD) [9]. Participants were asked to respond to the stem question (“I think including students with ADHD in my physical education class would be …”). Three 7-point semantic differential scales were used for responses: “extremely harmful” (1) to “extremely beneficial” (7), “extremely bad” (1) to “extremely good” (7), and “extremely worthless” (1) to “extremely useful” (7). The scale showed good internal reliability with the current sample (α = 0.91).

#### 2.2.3. Self-Esteem

We used the validated Chinese version of the Rosenberg Self-Esteem Scale to assess participants’ global self-esteem [22]. The scale has 10 items (e.g., “I feel that I am a person of worth, at least on an equal place with others”). Participants rated the items on a 4-point Likert scale that ranged from “strongly disagree” (1) to “strongly agree” (4). In the present study, the items demonstrated good internal reliability (α = 0.85).

#### 2.2.4. Self-Efficacy

We employed the validated Chinese version of the Physical Educators’ Self-Efficacy Toward Including Students with Disabilities–Autism [20] to measure participants’ self-efficacy toward teaching students with autism. The scale consists of 10 items (e.g., “Modify instructions for students with autism who are included in my general physical education class”). Participants responded on an 11-point Likert scale ranging from “cannot do at all” (0) to “highly certain can do” (10). The scale had excellent internal reliability with our sample (α = 0.93).

#### 2.2.5. Life Satisfaction

We used the validated Chinese version of the Satisfaction with Life Scale to measure participants’ life satisfaction [23]. The scale has five items (e.g., “The conditions of my life are excellent”). Participants rated the items on a 7-point Likert scale, which ranged from “strongly disagree” (1) to “strongly agree” (7). The scale showed good internal reliability with the current sample (α = 0.90).

### 2.3. Procedures

Ethical approval for this study was obtained from the Human Research Ethics Committee of The Education University of Hong Kong (Ref. no. 2015-2016-0370). Sample 1 was recruited through Facebook and emailing lecturers to invite their students (in-service physical education teachers) who were enrolling in professional development workshops. Accordingly, participants completed a paper-and-pencil-based survey form in a classroom or online after informed consent was collected. Sample 2 was recruited from five public universities. Participants who had part-time school teaching experience or had completed their teaching practice were invited by their course lecturers to complete a paper-and-pencil-based survey form. Written informed consent was collected, followed by the completion of the questionnaire in a classroom. Both samples spent about 10 min to complete the survey forms. Due to the mixed mode of survey administration, we were not able to calculate the response rate for Sample 1. The response rate for Sample 2 was 92.3%. In order to examine the stability of the MTS-C scores, a subgroup of Sample 2 (*n* = 46) was invited to complete the MTS-C within a month interval. Of those participants, 35 (76.1%) provided the responses.

### 2.4. Data Analyses

To examine the underlying structure of the MTS-C, Sample 1 was subjected to EFA with principal axis factoring using IBM’s SPSS 25 (Armonk, NY, USA). Promax rotation was used as the hypothesized factors would be correlated [17]. Both the Scree plot and factor interpretability were considered for determining the number of factors to be retained. Item factor loadings greater than 0.3 was deemed adequate. In addition, an item having a factor loading of 0.32 or higher on more than two factors was considered as a cross-loading item [24].

To cross-validate the factor structure derived from EFA, we conducted CFA with Sample 2. A maximum likelihood estimation procedure was followed and the analysis was conducted using M*plus* 7.0 (Muthén & Muthén, Los Angeles, CA, USA) [25]. Multiple fit indices were used to evaluate the model fit: comparative fit index (CFI), Tucker–Lewis index (TLI), and root mean square error of approximation (RMSEA). CFI/TLI values greater than 0.90 and a RMSEA value less than 0.08 are considered acceptable [26,27].

Following CFA, zero-order correlation coefficients were computed to investigate the relationships between the MTS-C and theoretically relevant constructs across both samples. Internal reliability of the MTS-C was also computed. Finally, intraclass correlation coefficients (ICC) were used to determine the test–retest reliability of the MTS-C among the subsample of Sample 2. An ICC value higher than 0.60 indicates good test–retest reliability [28]. These follow-up analyses were conducted using IBM’s SPSS 25.

## 3. Results

### 3.1. EFA (Sample 1)

The value of Kaiser–Meyer–Olkin (0.80) and the significant result of Bartlett’s test of sphericity (*p* < 0.001) supported the sampling adequacy of EFA. The initial analysis yielded a four-factor solution (eigenvalues of 1.05 to 4.24), explaining 59.62% of the total variance. The pattern matrix showed that factor 3 had four items loading on it (items 1, 2, 3, and 5) and two of them (items 1 and 5) cross-loaded on factor 1. In addition, factor 4 included three items (items 3, 7, and 9) and two of which (items 3 and 7) had substantial cross loadings on factor 1. Thus, we decided to rerun the analysis using the two-factor solution. The two-factor model (eigenvalues of 4.24 and 1.74) explained 42.72% of the total variance. Specifically, nine items loaded on Teaching Intrapersonal Mindfulness and accounted for 30.28% of the total variance. The five remaining items of Teaching Interpersonal Mindfulness accounted for 12.44% of the total variance. The two factors were positively correlated with each other (*r* = 0.39, *p* < 0.01). All item factor loadings were higher than 0.30 (see Table 1).

### 3.2. CFA (Sample 2)

The Sample 2 data showed an acceptable fit to the two-factor measurement model: χ^2^ (76) = 154.99, CFI = 0.906, TLI = 0.887, RMSEA = 0.068, 90% CI (0.052, 0.083). The modification index suggested that the error terms between items 13 and 14 could be correlated (modification index of 17.10). By also considering the item contents (see Table 1), the model was re-specified by correlating the two error terms. The re-specified model showed a better fit: χ^2^ (75) = 137.77, CFI = 0.925, TLI = 0.909, RMSEA = 0.061, 90% CI (0.044, 0.076). The two factors were not significantly correlated (*r* = 0.04, *p* = 0.62). All factor loadings were above 0.30 (see Table 1).

### 3.3. Concurrent Validity (Samples 1 and 2)

The results of concurrent correlations for the MTS-C subscales and their theoretically relevant measures are presented in Table 2. Intrapersonal mindfulness had a positive association with attitudes, self-esteem, self-efficacy, and life satisfaction (*r* = 0.21 to 0.42, *p* < 0.01) in Samples 1 and 2. In addition, interpersonal mindfulness was positively related to attitudes (*r* = 0.39, p < 0.01) and self-esteem (*r* = 0.35, *p* < 0.01) in Sample 2. However, interpersonal mindfulness was not associated with self-efficacy (*r* = −0.01, *p* = 0.87) and life satisfaction (*r* = −0.08, *p* = 0.25) in Sample 2. 

### 3.4. Reliability (Samples 1 and 2)

The two mindfulness subscales demonstrated adequate internal reliability across both samples (α = 0.71 to 0.84; Table 2). We also found good test–retest reliability of teaching intrapersonal mindfulness (ICC = 0.84) and teaching interpersonal mindfulness (ICC = 0.85) in the subgroup of Sample 2.

## 4. Discussion

In an effort to extend the use of the MTS to other populations, the present study examined the psychometric properties of the MTS among Chinese teachers. Specifically, we examined the factorial validity, concurrent validity, internal reliability, and test–retest reliability of the MTS-C in both pre-service and in-service Chinese teachers. In general, our analyses showed that the MTS-C is a valid and reliable measure. Another significant contribution of this study is the inclusion of pre-service teachers as a sample because only in-service teachers were recruited in earlier validation studies [6,17,18]. Pre-service teachers usually need to enroll in teaching practice or related training programs. Our results provide supporting evidence regarding the utility of the MTS-C in pre-service teachers. The MTS-C may therefore be used in mindfulness research and practice of pre-service teachers. 

In line with previous validation studies of the MTS, MTS-K, and MTS-T [6,17,18], the MTS-C was found to have the same two-factor structure. Similar to those earlier validation research studies, our study also found a moderate and positive association between the two factors among in-service teachers. Somewhat unexpectedly, these two factors were not significantly correlated with each other in the sample of pre-service teachers. This result could be sample specific, which warrants future investigations. Another possibility could be that, compared with in-service teachers, pre-service teachers have less experiences in student–teacher interactions. The interpersonal mindfulness subscale may therefore not be sensitive enough to gauge their attention to others and awareness of their own experiences in teaching.

In relation to concurrent validity, we found that the intrapersonal mindfulness subscale had a positive association with attitudes, self-esteem, self-efficacy, and life satisfaction. These findings are aligned with previous study findings, which reported that teachers higher in mindfulness showed more positive teaching attitudes, self-esteem, self-efficacy, and life satisfaction [6,20,29]. In terms of the interpersonal mindfulness subscale, it only had a significant association with attitudes and self-esteem. Its association with the other two criterion variables was not significant in our study. These findings suggest intrapersonal mindfulness may be more important to dimensions of attitudes and self-esteem than self-efficacy and life satisfaction. Taken together, our findings generally support the concurrent validity of the MTS-C. In addition, intrapersonal mindfulness seems to have a unique contribution to understanding teacher-related outcomes.

The internal reliability of the MTS-C subscales was adequate and comparable to that of the MTS [17]. Of note, internal consistencies of the interpersonal mindfulness were slightly below the traditional cut-off (α = 0.70) among Korean and Turkish samples [6,18]. As this subscale has five items, it is unlikely that the number of items is the major factor contributing to the relatively low internal reliability. Future research may need to examine this issue. With regard to test–retest reliability, we found that the MTS-C scores were relatively stable across one month. This finding is similar to early research, in which a high degree of temporal stability across six-month and three-week intervals was evident [17,18].

### Limitations and Future Research Directions

The present study has several limitations which should be noted. First, our samples are confined to primary and secondary school physical education teachers, and most of them are males. Other samples with balanced genders such as language teachers from kindergarten and university can be recruited in future validation studies. Second, although it is important to examine the psychometric properties of the MTS-C among pre-service teachers, some of the scale items may not be sensitive enough. Future validation research with pre-service teachers may need to revise some of the existing items or generate new items. Third, the sample sizes are too small to conduct multi-group CFA. With larger sample sizes, future studies can examine whether MTS-C scores are invariant across different groups (e.g., males vs. females) using multi-group CFA. Fourth, as a cross-sectional design was used, the causal relations among studied variables cannot be inferred. A longitudinal survey design can be used to examine the predictive validity of the MTS on outcomes such as teacher well-being and students’ academic performance. By applying the design, the longitudinal or time invariance of the MTS scores can also be examined. Finally, mindfulness-based intervention programs can be used to investigate the sensitivity of the MTS (i.e., sensitivity of the MTS is evident if participants reported increased MTS scores after the intervention).

## 5. Conclusions

In summary, scale development is an ongoing process and our findings provide cumulative evidence regarding the reliability and validity of the MTS. The MTS-C appears to have sound psychometric properties, including factorial validity, concurrent validity, internal reliability, and test–retest reliability among pre-service and in-service physical education teachers. The MTS-C may be therefore suitable for use in assessing Chinese teachers’ mindfulness in classroom settings by school stakeholders and health practitioners. It is hoped that our findings can spark more future studies on teacher mindfulness.

## Figures and Tables

**Table 1:** Items and factor loadings.

Item	EFA (Sample 1)		CFA (Sample 2)
	Intrapersonal	Interpersonal	
1. When I am teaching it seems I am “running on automatic” without much awareness of what I am doing. 当我在讲课时，我似乎是在“自动进行”教学，没有太多的意识到我正在做什么。	0.57		0.55
2. When I am in the classroom I have difficulty staying focused on what is happening in the present. 当我在课堂上时，我很难持续专注于当下发生的事情。	0.47		0.60
3. When I am teaching I find myself doing things without paying attention. 当我在讲课时，我发觉自己会做事心不在焉。	0.47		0.71
4. When I am teaching I get so focused on the goal I want to achieve that I lose touch with what I’m doing right now to get there. 当我在讲课时，我太专注于想要达到的教学目标，以至于忽略自己正用什么教学活动来达到目标。	0.71		0.66
5. At school I tend to walk quickly to get where I’m going without paying attention to what I experience along the way. 在学校里，我通常会一路直奔目的地，而没有注意到我沿途经历些什么。	0.75		0.53
6. I rush through activities with my class without being really attentive to them. 我仓促地完成教学活动，并没有真正地留意到它们。	0.75		0.77
7. When something painful happens at school I tend to blow the incident out of proportion. 当在学校里发生了一些不愉快的事情时，我倾向于将事态严重化。	0.62		0.56
8. I am often so busy thinking about other things that I am not really listening to my students. 我经常因为忙于思考其他的事情，以至于没有真正地聆听学生们对我说了什么。	0.69		0.57
9. When I’m really struggling with teaching, I tend to feel like other teachers must be having an easier time of it. 当我被教学所困扰而挣扎的时候，我通常会觉得其他老师遇到的教学困难一定比较少。	0.46		0.50
10. Even when it makes me uncomfortable, I allow my students to express their feelings. 我允许我的学生们表达他们的感受，即使有时这会使我感到不舒服。		0.76	0.65
11. I listen carefully to my student’s ideas, even when I disagree with them. 我会认真地聆听我的学生们的想法，即使有时我并不同意他们的观点。		0.78	0.73
12. I am aware of how my moods affect the way I treat my students. 我意识到我的心情如何影响我对待自己学生们的方式。		0.69	0.57
13. When I’m upset with my students, I notice how I am feeling before I take action. 当我对我的学生们感到心烦时，采取行动前我会注意到自己当下的情绪。		0.69	0.65
14. When I am upset with my class, I calmly tell them how I am feeling. 当我对我的学生们感到心烦时，我会冷静地告诉他们我当下的感受。		0.45	0.30

Note: For clarity, only factor loadings greater than 0.30 are presented. EFA, exploratory factor analysis; CFA, confirmatory factor analysis.

**Table 2:** Internal reliability and zero-order correlation between mindfulness and theoretically relevant constructs.

Variable (Sample)	Intrapersonal Mindfulness	Interpersonal Mindfulness
Attitudes (Sample 1)	0.40 **	0.39 **
Self-esteem (Sample 1)	0.42 **	0.35 **
Self-efficacy (Sample 2)	0.21 **	−0.01
Life satisfaction (Sample 2)	0.27 **	−0.08
α (Sample 1/2)	0.80/0.84	0.71/0.74

Note: ** *p* < 0.01.
